# Comparative Characterization of Shiga Toxin Type 2 and Subtilase Cytotoxin Effects on Human Renal Epithelial and Endothelial Cells Grown in Monolayer and Bilayer Conditions

**DOI:** 10.1371/journal.pone.0158180

**Published:** 2016-06-23

**Authors:** Romina S. Álvarez, Flavia Sacerdoti, Carolina Jancic, Adrienne W. Paton, James C. Paton, Cristina Ibarra, María M. Amaral

**Affiliations:** 1 Laboratorio de Fisiopatogenia, Departamento de Fisiología, Facultad de Medicina, Universidad de Buenos Aires, Buenos Aires, Argentina; 2 Laboratorio de Inmunidad Innata, Instituto de Medicina Experimental (IMEX-CONICET), Academia Nacional de Medicina, Buenos Aires, Argentina; 3 Research Centre for Infectious Diseases, Department of Molecular and Cellular Biology, University of Adelaide, Adelaide, Australia; Medical College of Wisconsin, UNITED STATES

## Abstract

Postdiarrheal hemolytic uremic syndrome (HUS) affects children under 5 years old and is responsible for the development of acute and chronic renal failure, particularly in Argentina. This pathology is a complication of Shiga toxin (Stx)-producing *Escherichia coli* infection and renal damage is attributed to Stx types 1 and 2 (Stx1, Stx2) produced by *Escherichia coli* O157:H7 and many other STEC serotypes. It has been reported the production of Subtilase cytotoxin (SubAB) by non-O157 STEC isolated from cases of childhood diarrhea. Therefore, it is proposed that SubAB may contribute to HUS pathogenesis. The human kidney is the most affected organ because very Stx-sensitive cells express high amounts of biologically active receptor. In this study, we investigated the effects of Stx2 and SubAB on primary cultures of human glomerular endothelial cells (HGEC) and on a human tubular epithelial cell line (HK-2) in monoculture and coculture conditions. We have established the coculture as a human renal proximal tubule model to study water absorption and cytotoxicity in the presence of Stx2 and SubAB. We obtained and characterized cocultures of HGEC and HK-2. Under basal conditions, HGEC monolayers exhibited the lowest electrical resistance (TEER) and the highest water permeability, while the HGEC/HK-2 bilayers showed the highest TEER and the lowest water permeability. In addition, at times as short as 20–30 minutes, Stx2 and SubAB caused the inhibition of water absorption across HK-2 and HGEC monolayers and this effect was not related to a decrease in cell viability. However, toxins did not have inhibitory effects on water movement across HGEC/HK-2 bilayers. After 72 h, Stx2 inhibited the cell viability of HGEC and HK-2 monolayers, but these effects were attenuated in HGEC/HK-2 bilayers. On the other hand, SubAB cytotoxicity shows a tendency to be attenuated by the bilayers. Our data provide evidence about the different effects of these toxins on the bilayers respect to the monolayers. This *in vitro* model of communication between human renal microvascular endothelial cells and human proximal tubular epithelial cells is a representative model of the human proximal tubule to study the effects of Stx2 and SubAB related to the development of HUS.

## Introduction

Shiga toxin (Stx)-producing *Escherichia coli* infection is responsible for the development of hemolytic uremic syndrome (HUS) [1], characterized by non-immune hemolytic anemia, thrombocytopenia and acute renal failure (ARF) [2].

In Argentina, postdiarrheal HUS is endemic and over the last 10 years, approximately 400 new cases were reported annually. The incidence ranged from 10 to 17 cases per 100,000 children less than 5 years of age, and the lethality was between 1 and 4% [3]. HUS is highly prevalent in Argentina being the most common cause of ARF and the second leading cause of chronic renal failure (CRF) in children younger than 5 years old [4, 5].

Stx type 1 and type 2 (Stx1 and Stx2), produced by STEC O157:H7 and non-O157:H7 strains are considered the main virulence factors that trigger the renal damage in HUS patients. STEC strains expressing Stx2 are mainly responsible for severe cases of HUS in Argentina [6]. Both types of toxins and their allelic variants are encoded in bacteriophages integrated in the STEC genome [7].

The risks of infection by STEC are related to host factors, reservoirs, as well as biological and cultural factors of the host. Humans can become infected by ingestion of inadequately cooked meat products, vegetables, unpasteurized dairy products contaminated with STEC. They can also be infected by drinking or swimming in contaminated water, direct contact with animals and transmission from person to person by the fecal-oral route, favored by the low infectious dose of STEC (<100 bacteria per gram of food) [8].

After bacteria are ingested, these pathogens colonize the bowel and release Stx into the lumen of the gut. Then, Stx can access the systemic circulation and reaches the plasma membrane of target cells and binds the glycolipid globotriaosylceramide (Gb3) [9]. Stx is internalized into the cell by a receptor mediated endocytosis and the toxin goes to a retrograde transport to the Golgi network and endoplasmic reticulum (ER) where the A subunit is cleaved in two fragments A1 and A2. A1 is then translocated to the cytosol where it exhibits its ribosome-inactivating activity that leads to protein synthesis inhibition and the activation of cell stress response pathways that trigger the apoptosis [10]. In this regard, the stress elicited by the inactivated ribosomes induces multiple stress associated signaling pathways. The ribotoxic stress response is activated and this stress leads to activation of Mitogen-activated protein kinases (MAPK) signaling pathways critical for innate immunity activation and apoptosis regulation [10]. Stx comprise a single 30 kDa A-subunit and a pentamer of noncovalently attached identical 7 kDa B-subunits. Enzymatic activity resides in the A subunit whereas the cell recognition receptor binding properties are in the B-subunits [11].

Subtilase (SubAB) is a cytotoxin produced by virulent STEC strains which are negative for the locus of enterocyte effacement [12–15]. *E*. *coli* O157:H7 is the most prevalent serotype associated with HUS, although non-O157 STEC including LEE negative strains predominate in Argentina, where HUS incidence is the highest in the world [13]. SubAB was first described in a strain of STEC belonging to serotype O113:H21, responsible for an outbreak of HUS in Australia [16] and also isolated in Argentina [17]. SubAB cytotoxicity on eukaryotic cells involves a proteolytic cleavage of the chaperone BIP (GRP78) [18] that triggers a massive ER response and finally promotes apoptosis [19–21]. Although the specific receptor for SubAB has not been described, it has been postulated that this cytotoxin binds glycans terminating in N-glycolylneuraminic acid (Neu5Gc) [22] and this monosaccharide is considered the key component of SubAB receptors. Humans cannot synthesize Neu5Gc but it can be incorporated into human tissues by dietary intake. It has been described that Vero cells [23] and HeLa cells [24] express SubAB-binding proteins containing Neu5Gc. Direct action of SubAB on the cell viability decrease, associated with apoptosis, was observed in different cell types, in particularly on human renal cells such as human glomerular endothelial cells (HGEC) and human renal tubular epithelial cells (CERH) [25, 26]. In addition, SubAB-treated mice show the HUS-like pathological features [27]. The contribution of SubAB to HUS physiopathology is still unknown, but several authors postulated it as a potential cytotoxin to augment clinical manifestations of STEC infection [13]. Although, SubAB has not been detected in patients’ blood, several STEC serotypes expressing SubAB have been related to HUS cases around the world [17].

The kidney is the most affected organ in postdiarrheal HUS, where very Stx-sensitive cells express high amounts of Gb3 [28]. This receptor has been described in human microvascular endothelial cells [25], proximal tubule epithelial cells, mesangial cells, podocytes, and others [29, 30]. Previously, we developed primary cultures of HGEC and demonstrated that Stx2 decreased cell viability by endothelial injury similarly to that documented in biopsies of HUS patient kidneys. In addition, Gb3 mediates Stx2 cytotoxic effects in these cells [25]. Recently, we have shown that a human proximal tubular epithelial cell line (HK-2) is sensitive to Stx2 and this fact is related to Gb3 receptor expression [31]. Earlier, it has been suggested that renal tubular injury observed in HUS patients [32] is induced as a consequence of the damage caused on glomeruli and arterioles and also due to a direct effect of Stx on the tubules [33]. However, there is little information in the literature with respect to the effect of endothelial dysfunction mediated by Stx on renal tubular epithelial function. There is evidence to support the hypothesis that renal microvascular endothelial cells and proximal tubular cells cooperate in solute and water re-absorption and secretion [34]. In addition, the microvasculature can promote immune cells migration to damaged tubules [35]. Taking into account these antecedents, our aim was to investigate the effects of Stx2 and SubAB on endothelial and epithelial cells using a filter-based, noncontact, close proximity coculture of HGEC and HK-2. In this study, we have established the coculture as a human renal proximal tubule model to study water absorption and cytotoxicity in the presence of Stx2 and SubAB. The data described here show that Stx2 and SubAB effects are different when monoculture and coculture were compared. These results will be important in the further elucidation of endothelial and epithelial cross talk mechanisms involved in the toxins’ action on kidney cells.

## Materials and Methods

### Reagents

Purified Stx2a was provided by Phoenix Laboratory, Tufts Medical Center, Boston, MA, USA. SubAB was purified from recombinant *E*. *coli* by Ni-NTA chromatography via a His_6_ tag fused to the C-terminus of the B subunit, as described previously [16]. Purity was greater than 98%, as judged by SDS-PAGE and staining with Coomassie Blue.

### Primary culture

Human glomerular endothelial cells (HGEC) were isolated from kidneys fragments removed from normal areas from different pediatric patients with segmental uropathies or tumor in one pole and normal creatinine that were undergoing nephrectomies performed at Hospital Nacional “Alejandro Posadas”, Buenos Aires, Argentina (written informed consent was obtained from the next of kin, caretakers, or guardians on the behalf of the minors/children participants involved in our study). The Ethics Committee of the University of Buenos Aires approved the use of human renal tissues for research purposes. Endothelial cells were isolated as was previously described [25]. For growth-arrested conditions, a medium with a half of the FCS concentration (10%) and without endothelial cell growth supplement (ECGS) was used. For the experiments, cells were used between 2–7 passages, after characterization for von Willebrand factor (VWF; DAKO, Tecnolab, Argentina) and platelet/endothelial cell adhesion molecule 1 (PECAM-1, DAKO, Tecnolab, Argentina) positive expression [25].

### Cell line culture

Human proximal tubular epithelial cell line (HK-2) was purchased from American Type Culture Collection (ATCC, Manassas, VA) and grown in DMEM/F12 medium (Sigma Aldrich, USA) containing 10% FCS, 100 U/ml penicillin/streptomycin (GIBCO, USA), 2 mM L-glutamine, 15 mM HEPES at 37°C in a humidified 5% CO_2_ incubator. For growth-arrested conditions, medium without FCS was used.

### Experimental design

#### Renal endothelium-epithelium monoculture and coculture construction

**Fig 1:**
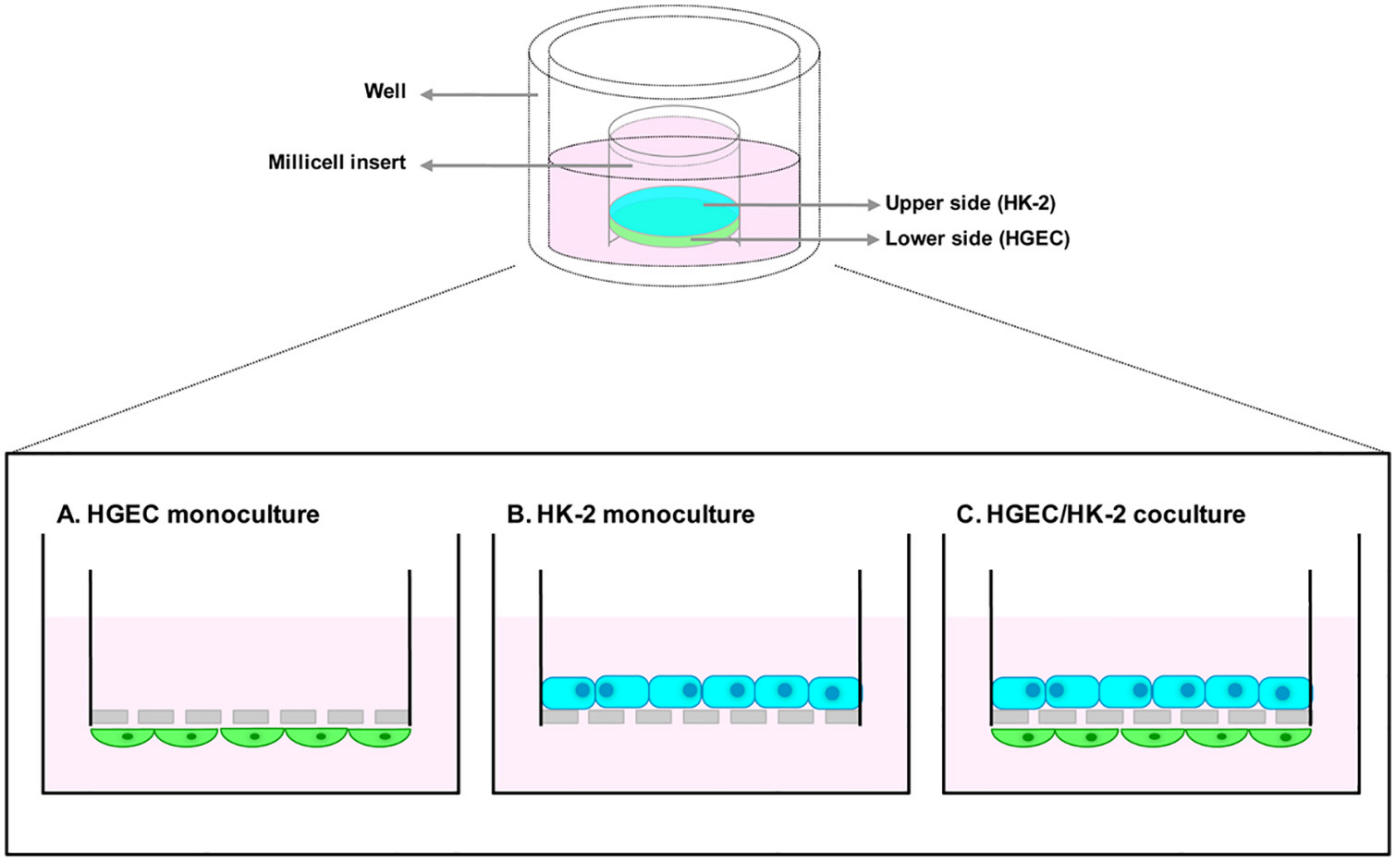
Schematic description of cell monoculture and coculture systems. HGEC cells were seeded on the lower side of a Millicell support (0.4 μm membrane pore size), and HK-2 cells into the upper side. Cells were either cultured in monoculture (**A** and **B**) or in coculture (**C**). To evaluate the effects of Stx2 and SubAB on monoculture and coculture, Stx2 or SubAB were added to the lower side.

Cocultures of HGEC and HK-2 cells were performed using Millicell cell culture inserts (PIHP01250, Millipore, Billerica, MA, USA). HGEC cells (5.10^4^) were seeded on the lower side of the filter (0.4 μm membrane pore size) and allowed to attach for 12–16 hours. Then, inserts were reverted and HK-2 (7.10^4^) cells were seeded into the upper side (Fig 1). Bilayers were maintained in HGEC complete medium. For the epithelial and endothelial monocultures, the same procedure was carried out with the exception that partner cells were not added. The bilayer formation was observed by optical and fluorescence microscopy in paraffin cuts stained with H&E and Hoechst.

### Measurement of electrical resistance

The electrical resistance (TEER) across the monolayers and bilayers was measured with a Millicell-ERS electric resistance system (Millipore, Billerica, MA, USA) calibrated for each measurement. TEER is accepted to measure the integrity of tight junction in cell culture models of endothelial and epithelial monolayers [36]. TEER expressed as Ω.cm^2^ (filter area: 1.13 cm^2^) was monitored daily during the development of cell culture until confluence was achieved. Data were corrected for the resistance measurements across blank inserts that showed a resistance of 161.7 ± 2.1 Ω.cm^2^ (n = 6).

### Net water transport (Jw) measurement

To evaluate the net water transport (Jw), HK-2 were placed on the upper side and HGEC were cultured in the lower side of the filters. In this way, it is possible to apply a hydrostatic pressure on the upper side when the filters are inserted in a modified Ussing chamber. So, net water transport occurs from HK-2 cells (upper side) to HGEC (lower side), representing the reabsorption of water that takes place in the renal proximal tubule.

Jw was recorded automatically across the monolayers and bilayers inserted in an Ussing chamber connected to a special electro-optical device as was previously described [37]. Briefly, to perform the Jw measurements, confluent cell monolayers or bilayers grown on Millicell filters were directly inserted into a chamber slider for the Ussing chamber (Harvard Apparatus, USA) and filled with standard Ringer solution containing (in mM): 113 NaCl, 4.5 KCl, 25 NaHCO_3_; 1.2 MgCl_2_; 1.2 CaCl_2_; 1.2 K_2_HPO_4_; 0.2 KH_2_PO_4_; 25 glucose. The endothelial side was continuously bubbled with 95% O_2_−5% CO_2_, and the cell temperature was kept at 37°C by a water-jacket reservoir connected to a constant temperature circulating pump. The epithelial side was closed, and a hydrostatic pressure of 4.5 cm of H_2_O for HGEC or 7 cm of H_2_O for HK-2 and HGEC/HK-2, was continuously applied on this side. Water movement across the monolayers and bilayers was measured by displacement of a photo-opaque solution inside a glass capillary tube connected to the upper side of the chamber via an intermediate chamber. The liquid meniscus movement in the glass capillary was detected using an electro-optical device connected to a computer [38]. The sensitivity of this instrument is approximately 50 nl. When the parameters were stabilized, Stx2 (10 ng/ml), SubAB (1500 ng/ml) or PBS (control) was added to the endothelial (lower) side (time 0). Then, Jw was recorded every minute for 50 min. Because of cell variability, data were analyzed as ΔJw where ΔJw = Jw (at a given time)—Jw (at time 0).

### Neutral red cytotoxicity assay

The neutral red cytotoxicity assay was adapted from previously described protocols [39]. To evaluate the Stx2 and SubAB effect on viability, HGEC and HK-2 monolayers as well as HGEC/HK-2 bilayers were treated with Stx2 (1ng/ml) and SubAB (150 ng/ml) in growth-arrested conditions. After 72 h of treatment, freshly diluted neutral red (Sigma Aldrich, USA) was added in a final concentration of 10 μg/ml and then cells were incubated for an additional 1 h at 37°C in 5% CO_2_. Cells were then washed and fixed with 200 μl 1% CaCl_2_ + 1% formaldehyde and then lysed with 200 μl 1% acetic acid in 50% ethanol to solubilized the neutral red. Absorbance in each well was measure in an automated plate spectrophotometer at 540 nm. Results were expressed as percentage of viability, where 100% represents cells incubated under identical conditions but without toxins.

### Data analysis

Data are presented as mean ± SEM. Statistical analysis was performed using the Graph Pad Prism Software 5.0 (San Diego, CA, USA). ANOVA was used to calculate differences between groups and Tukey’s multiple comparisons test was used as an a posteriori test. Statistical significance was set at *P* < 0.05.

## Results

### Morphology of coculture system

**Fig 2:**
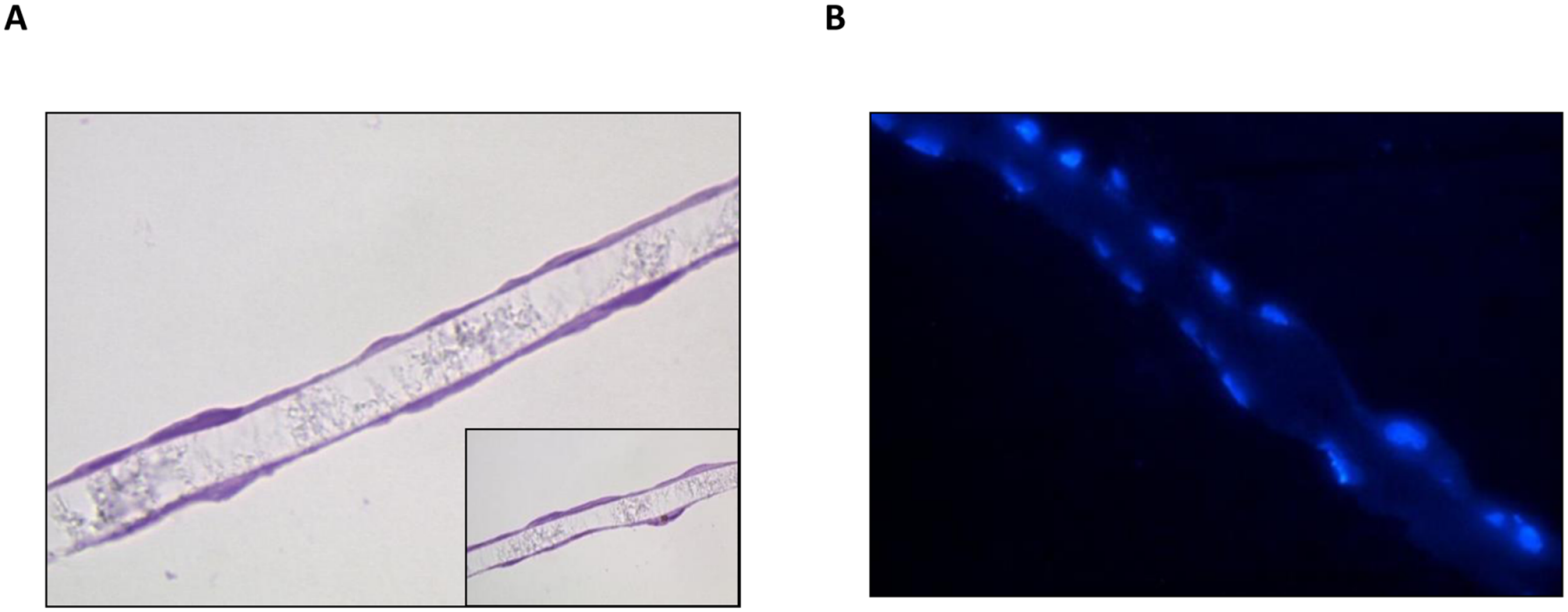
Morphology of HGEC/HK-2 bilayer. Human glomerular endothelial cells (HGEC) and human proximal tubular epithelial cell line (HK-2) were grown in Millicell inserts as described. After confluence (7 days of culture), the filters were fixed, sectioned and stained with H&E (**A**) or Hoechst (**B**) to be observed by optical and fluorescence microscopy, respectively. **A** and **B** (**×**400); insert in panel **A** (**×**1000 magnification).

HGEC and HK-2 cells were grown in Millicell inserts until confluence, processed and stained with H&E (Fig 2A) and Hoechst (Fig 2B). Both, optical and fluorescence microscopy showed a monolayer of adhered cells stained with H&E and Hoechst, respectively, on both sides of the filter.

### Integrity of endothelial and epithelial monolayers and bilayers

**Fig 3:**
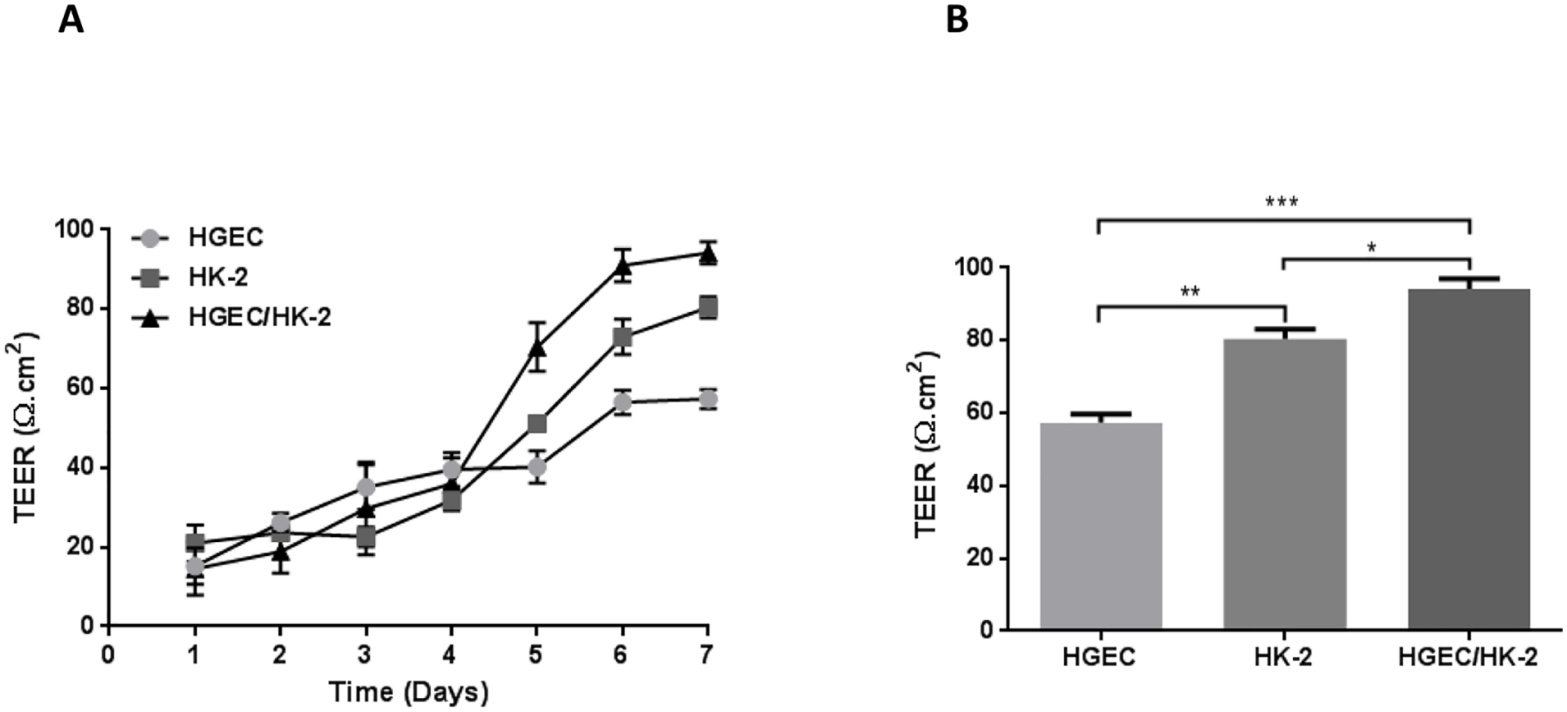
Integrity of endothelial and epithelial monolayers and bilayer. (**A**) The electrical resistance (TEER, Ω.cm^2^) across monolayers and bilayers was measured during the development of cell culture. (**B**) Differences in TEER values between HGEC and HK-2 monlayers and HGEC/HK-2 bilayer at confluence (7 days). Each value represents mean ± SEM, of six experiments. *P <0.05, **P <0.001, ***P <0.0001.

Integrity of endothelial and epithelial monolayers and bilayers was checked by measuring TEER values until confluence. As shown in Fig 3A, monolayers and bilayers showed a time-dependent increase in the TEER from the 1^st^ to the 6^th^ and 7^th^ days after beginning cell culture. At this time, TEER values were stabilized, indicating that HGEC and HK-2 monolayers and HGEC/HK-2 bilayers had reached confluence. As expected, HGEC showed TEER values significantly lower than HK-2. On the other hand, HGEC/HK-2 bilayers showed higher TEER values than HGEC and HK-2 monolayers (HGEC/HK-2: 94.2 ± 3.0 Ω cm^2^ *vs* HGEC: 57.4 ± 2.4 Ω cm^2^ and HK-2: 82.4 ± 3.0 Ω cm^2^, n = 6) (Fig 3B).

### Functional characterization

Functional characterization of HGEC and HK-2 monolayers and HGEC/HK-2 bilayers was evaluated by assaying Jw.

**Fig 4:**
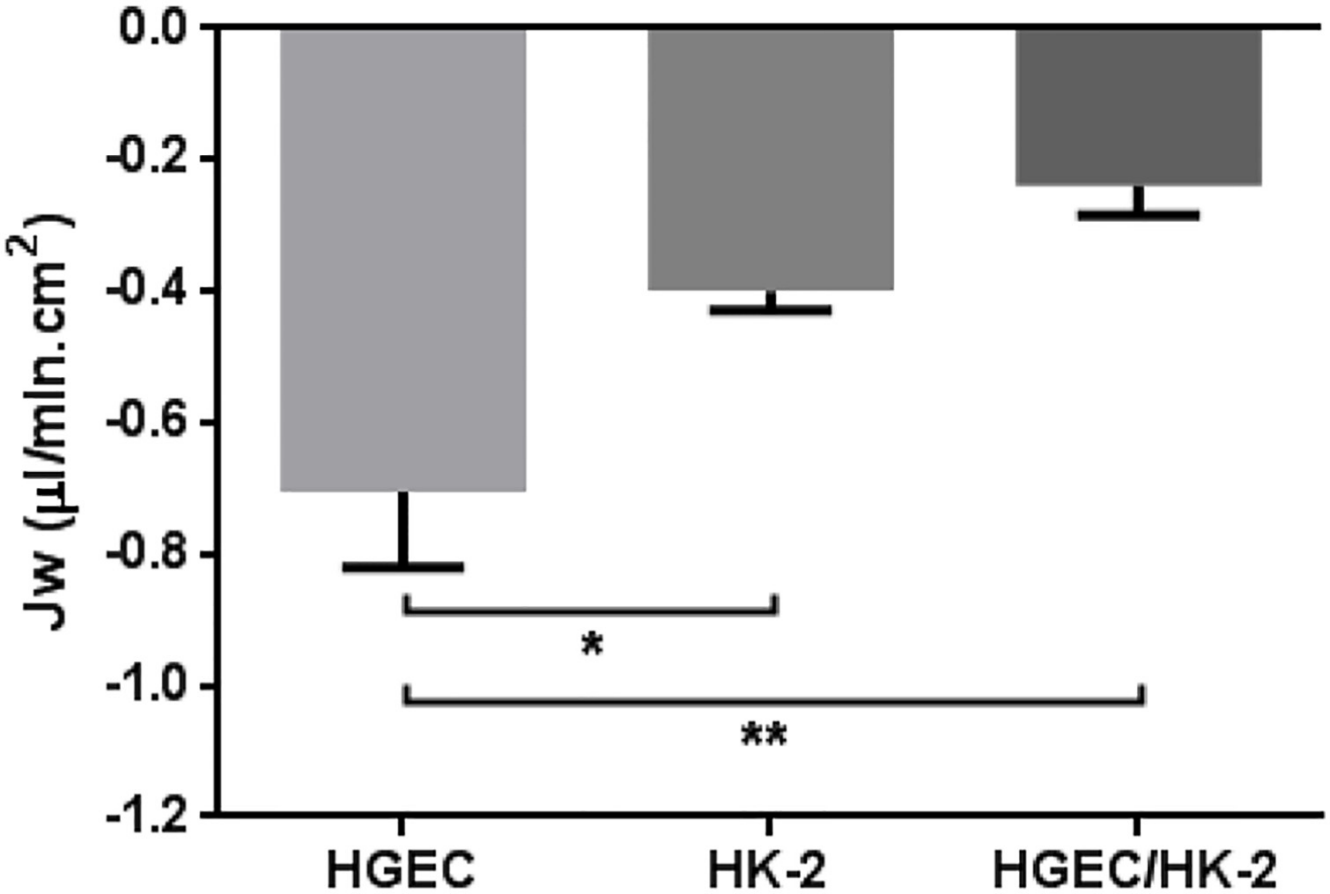
Functional characterization of monolayers and bilayers. Under basal conditions, the net absorptive water transport (Jw, μl/min.cm2) was recorded in HGEC, HK-2 monolayers and HGEC/HK-2 bilayer. Each value represents mean ± SEM, of six experiments. *P 0.05, **P< 0.001.

Under basal conditions, a net absorptive Jw (μl/min.cm^2^) was observed in monolayers and bilayers. As shown in Fig 4, the net absorptive Jw across HGEC monolayers was significantly higher than Jw measured in HK-2 monolayers and HGEC/HK-2 bilayers (HGEC: -0.71 ± 0.11*vs* Jw; HK-2: -0.40 ± 0.02 and Jw; HGEC/HK-2: -0.24 ± 0.04 Jw, n = 6) suggesting that water absorption is dependent on the cell type and culture conditions.

### Cytotoxic effects of Stx2 and SubAB on Jw

**Fig 5:**
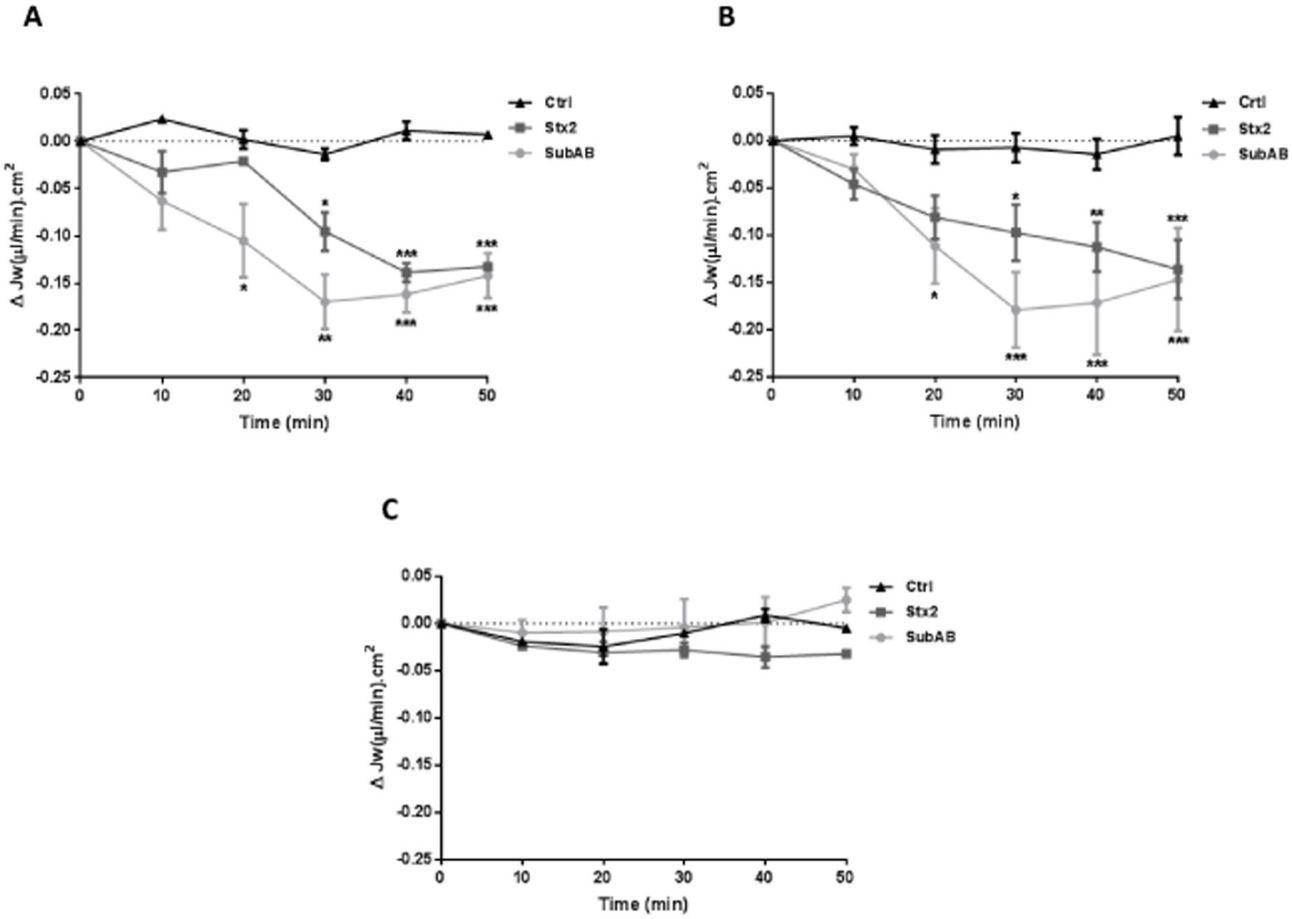
Effects of Stx2 and SubAB on the net absorptive water transport (Jw). Data represent the time course of the Jw across HGEC (A), HK-2 (B) and HGEC/HK-2 (C) incubated with PBS (Ctrl) or Stx2 (10 ng/ml) or SubAB (1500 ng/ml) (time = 0) on the lower side. A time-dependent Jw inhibition was observed in the case of monolayers but not in the bilayer. Each value represents mean ± SEM, of three experiments. Stx2 or SubAB vs Ctrl, *P <0.05, **P <0.001, ***P <0.0001.

Stx2 (10 ng/ml) or SubAB (1500 ng/ml) was added to the lower side (t = 0) of HGEC (Fig 5A) and HK-2 (Fig 5B) monolayers and HGEC/HK-2 bilayers (Fig 5C). Stx2 caused a significant inhibition of Jw relative to PBS controls in HGEC and HK-2 cells after 30 min. Unlike for monolayers, Stx2 did not have any significant inhibitory effect on the Jw across HGEC/HK-2 bilayers at any time.

For SubAB, a significant inhibition of Jw relative to PBS controls in HGEC and HK-2 cells was observed after 20 min of incubation with the toxin. Similar to Stx2, SubAB did not have any inhibitory effect on the Jw across HGEC/HK-2 bilayers at any time.

In order to evaluate if the inhibition of Jw could be related to a decrease in the cell viability, HGEC and HK-2 monolayers were treated with Stx2 or SubAB during short times (30, 60, 90 and 120 min). As shown in Table 1, toxins did not affect cell viability at any time evaluated.

**Table 1:** Effects of Stx2 or SubAB on cell viability at short time.

Viability (%)					
	control	30 min	60 min	90 min	120 min
Stx2					
HGEC	100.0	98.2 ± 5.0	93.5 ± 3.4	90.0 ± 3.6	93.0 ± 3.4
HK-2	100.0	100.0 ± 1.0	88.1 ± 2.4	90.8 ± 4.6	90.8 ± 4.6
SubAB					
HGEC	100.0	91.3 ± 6.6	94.1 ± 4.3	93.0 ± 5.4	91.0 ± 7.4
HK-2	100.0	97.0 ± 8.2	100.0 ± 12.0	96.0 ± 6.0	100.0 ± 1.0

### Inhibition of cell viability by Stx2 and SubAB

**Fig 6:**
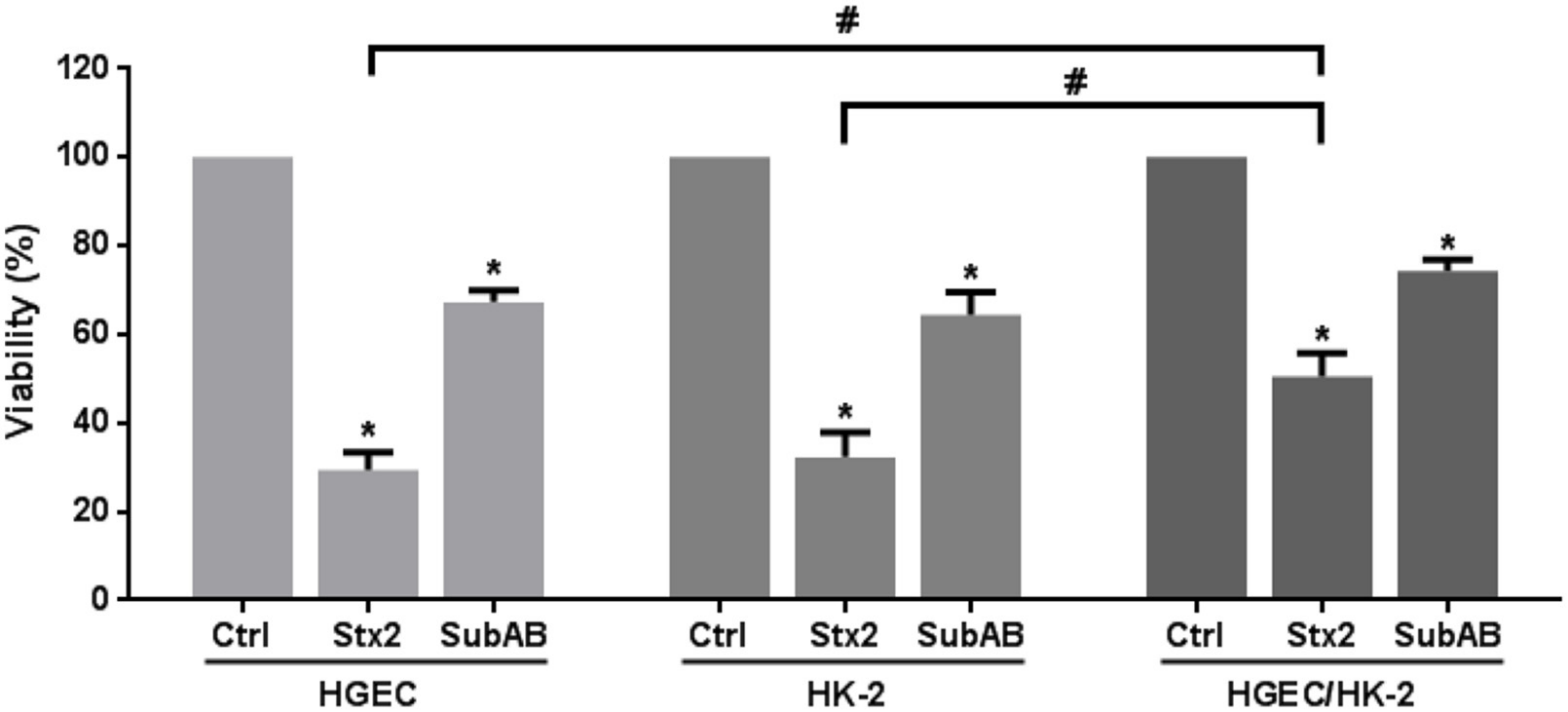
Inhibition of cell viability in monolayers and bilayer by Stx2 and SubAB. HGEC and HK-2 monolayers and HGEC/HK-2 bilayer were exposed to 1 ng/ml Stx2 or 150 ng/ml SubAB in growth-arrested conditions for 72 h. Then, cells were incubated with neutral red for an additional 1 h at 37°C in 5% CO_2_. Absorbance of each well was read at 540 nm. One hundred percent represents cells incubated under identical conditions but without toxin treatment (Ctrl). Results are expressed as means ± SEM of nine experiments, Stx2 or SubAB vs Ctrl, *P <0.05 and HGEC/HK-2 vs HGEC or HK-2, #P <0.05.

We compared the cytotoxic effects of Stx2 and SubAB on the cell viability of HGEC and HK-2 monolayers and HGEC/HK-2 bilayers. Before that, we studied the effect of SubAB on HK-2 and we found that after 72 h, a significant decrease in HK-2 viability was observed with SubAB at concentrations from 0.15 ng/ml to 15000 ng/ml. The range of SubAB concentrations (15 to 1500 ng/ml) represents approximately 50% cell lethality (**Data not shown**). A similar result was previously found in HGEC [25]. Then, HGEC and HK-2 monolayers and HGEC/HK-2 bilayers were treated with Stx2 (1ng/ml) or SubAB (150 ng/ml) and cell viability was measured at 72 h. Fig 6 shows that both toxins caused a significant inhibition of cell viability in HGEC and HK2 monolayers. In addition, Stx2 cytotoxicity was significantly attenuated when HGEC and HK2 were cocultured (HGEC/HK-2: 50.5 ± 5.4% *vs* HGEC: 29.4 ± 4.1% and HK-2: 32.3 ± 5.6%; n = 9). Even though it is not statistically significant, the viability of SubAB-treated cocultures is actually greater than that for monocultures (HGEC/HK-2: 74.25 ± 2.71% *vs* HGEC: 67.34 ± 2.66% and HK-2: 64.45 ± 5.10%; n = 9) (Fig 6).

## Discussion

Postdiarrheal HUS is the main cause of ARF in children and the second cause of CRF in Argentina [4, 5]. This pathology is not easily preventable and yet no effective treatment is known. Studies of HUS pathogenesis may identify new targets of therapeutic action to prevent or reduce the deleterious effects in organs such as the kidney. *In vitro* models have been focused on analyzing the effect of Stx and SubAB on monocultures of human renal cells. However, these models do not consider the communication that exists *in vivo* between renal cells. Until now, the effect of Stx2 and SubAB on cocultures of human renal endothelial and epithelial cells has not been investigated. This communication could modify the action of the toxins relative to that observed in monoculture. In this sense, Tasnim and Zink have demonstrated that human primary renal proximal tubular cells stimulated the endothelial cells to generate a special microenvironment of secreted soluble factors that improved their performance [40].

It is well known that proximal tubular epithelial cells and peritubular microvascular endothelial cells are in close proximity in the renal cortex. In this anatomical site, water and solutes are reabsorbed by the proximal tubule and then taken up to the microvasculature. In this sense, we have developed a coculture system to study water absorption and cytotoxicity in the presence of Stx2 and SubAB where human proximal tubular epithelial cells and human microvascular endothelial cells are in very close proximity. Our HEGC/HK-2 coculture system is an *in vitro* model to study toxins’ effects that attempts to simulate the *in vivo* human renal proximal tubular physiological function. We first characterized the integrity of bilayers composed of HGEC and HK-2. Correct formation of endo-epithelial bilayers was verified by the presence of adhered cells on both sides of a permeable support. We determined the integrity of endothelial or/and epithelial barriers by measuring the TEER across mono- and bilayers. TEER measurements showed an increase over days related to the cells growth and values were stabilized when the cells reached confluence. After stabilization, HGEC monolayers exhibited lower TEER values than HK-2 monolayers in agreement with previous reports [41, 42]. In addition, TEER values were higher in bilayers than monolayers indicating the influence of endothelial cells on epithelial cells. In this sense, TEER reflects the paracellular tightness of tight junctions that in “leaky” epithelia is responsible for the passage of proteins, ions, and water [43, 44]. Some studies have proposed that tight junctions of renal endothelial and epithelial cells have differences in the molecular composition that may contribute to defining the tightness of the intercellular junction [45]. In particular, the lability of tight junctions in the endothelium causes them to open and close to allow migration of leukocytes from the blood to the interstitial space [46].

Next, we characterized the functionality of bilayers by studying the net absorptive Jw. Under basal conditions, HGEC monolayers exhibited the highest net absorptive Jw compared to HK-2 monolayers, while HGEC/HK-2 bilayers had the lowest values of Jw. These results were coincident with TEER values obtained in monolayers and bilayers. While HGEC exhibited the lowest TEER and the highest water permeability, HGEC/HK-2 showed the highest TEER and the lowest water permeability.

In this work, we also observed the ability of Stx2 and SubAB to inhibit the net absorptive Jw across HGEC and HK-2 monolayers and this effect was not related to a decrease in cell viability. Both toxins were added to the endothelial side of monolayers and bilayers taking into account that if both toxins are released into the gut lumen after STEC colonization, they are absorbed into the circulation and have to cross the endothelial cells to damage the target cells [9, 10, 24, 27]. These results suggest that toxins could cause direct alterations in the mechanisms involved in the water transport across endothelial and/or epithelial monolayers as previously demonstrated for primary cultures of human renal epithelial cells [37]. In addition, toxins did not have inhibitory effects on water movement in HGEC/HK-2 bilayers indicating a protective effect caused by a close-proximity endothelium/epithelium. An alternative explanation is that water moves in a paracellular fashion crossing two sets of tight junctions in a bilayer. However, several authors have studied the influence of microvascular endothelial cells on function of epithelial cells. In this regard, Aydin *et al* identified a number of potential endothelium-derived factors and soluble growth factors that are most likely involved in the regulation of the renal epithelium [41]. Moreover, human proximal tubular cells stimulated their own performance by acting on endothelial cells [40].

Further, experiments showed that Stx2 and SubAB caused a significant inhibition of cell viability in HGEC and HK-2 monolayers after 72 h. While Stx2 effects were significantly attenuated in HGEC/HK-2 bilayers, SubAB effects evidenced a tendency to decrease in these coculture conditions. These results show again that damage produced in renal epithelial and endothelial *in vitro* are attenuated by a close-proximity coculture of HGEC and HK-2.

In line with our results, Bertocchi *et al* have shown that under coculture conditions interrelation between epithelial and endothelial cells appears to counteract the potentially harmful effects of epithelial NOS inhibition [47]. Nevertheless, Gomez *et al* have demonstrated that Stx2 caused an oxidative imbalance in an *in vivo* model. In this sense, the authors proposed that neutrophils would be the cells responsible for producing reactive oxygen species during Stx intoxication [48]. The activation of neutrophils will potentiate the inflammatory process involved in the acute renal failure characteristic of HUS [49]. In summary, we have demonstrated that the coculture of human renal microvascular endothelial cells and human proximal tubular epithelial cells is a representative *in vitro* model of the human proximal tubule anatomy and physiology to study the effects of Stx2 and SubAB on its functionality in the kidney. Our results show that toxins effects on bilayers are different from those observed on monolayers. In this sense, we can speculate that soluble mediators released from endothelial and/or epithelial cells could be involved in these different toxins effect. Future studies will be focused to study the possible soluble mediators implicated in these differences. Furthermore, the data described here will be important in the further elucidation of other multiple bacterial and inflammatory host components that may define the course of STEC infection.
